# The Current Status and Challenges in the Development of Vaccines and Drugs against Severe Acute Respiratory Syndrome-Corona Virus-2 (SARS-CoV-2)

**DOI:** 10.1155/2021/8160860

**Published:** 2021-06-01

**Authors:** Narasimha M. Beeraka, SubbaRao V. Tulimilli, Medha Karnik, Surya P. Sadhu, Rajeswara Rao Pragada, Gjumrakch Aliev, SubbaRao V. Madhunapantula

**Affiliations:** ^1^Center of Excellence in Molecular Biology and Regenerative Medicine (CEMR), Department of Biochemistry, JSS Academy of Higher Education & Research (JSS AHER), Mysore, 570015 Karnataka, India; ^2^Sechenov First Moscow State Medical University (Sechenov University), St. Trubetskaya, 8, Bld. 2, Moscow 119991, Russia; ^3^AU College of Pharmaceutical Sciences, Andhra University, Visakhapatnam, 530003 Andhra Pradesh, India; ^4^Institute of Physiologically Active Compounds, Russian Academy of Sciences, Chernogolovka, Moscow Region 142432, Russia; ^5^Research Institute of Human Morphology, 3Tsyurupy Street, Moscow 117418, Russia; ^6^GALLY International Research Institute, 7733 Louis Pasteur Drive, #330, San Antonio, TX 78229, USA; ^7^Special Interest Group in Cancer Biology and Cancer Stem Cells (SIG-CBCSC), JSS Medical College, JSS Academy of Higher Education & Research (JSS AHER), Mysore, 570015 Karnataka, India

## Abstract

Severe acute respiratory syndrome-coronavirus-2 (SARS-CoV-2) infection causes coronavirus disease-19 (COVID-19), which is characterized by clinical manifestations such as pneumonia, lymphopenia, severe acute respiratory distress, and cytokine storm. S glycoprotein of SARS-CoV-2 binds to angiotensin-converting enzyme II (ACE-II) to enter into the lungs through membrane proteases consequently inflicting the extensive viral load through rapid replication mechanisms. Despite several research efforts, challenges in COVID-19 management still persist at various levels that include (a) availability of a low cost and rapid self-screening test, (b) lack of an effective vaccine which works against multiple variants of SARS-CoV-2, and (c) lack of a potent drug that can reduce the complications of COVID-19. The development of vaccines against SARS-CoV-2 is a complicated process due to the emergence of mutant variants with greater virulence and their ability to invoke intricate lung pathophysiology. Moreover, the lack of a thorough understanding about the virus transmission mechanisms and complete pathogenesis of SARS-CoV-2 is making it hard for medical scientists to develop a better strategy to prevent the spread of the virus and design a clinically viable vaccine to protect individuals from being infected. A recent report has tested the hypothesis of T cell immunity and found effective when compared to the antibody response in agammaglobulinemic patients. Understanding SARS-CoV-2-induced changes such as “Th-2 immunopathological variations, mononuclear cell & eosinophil infiltration of the lung and antibody-dependent enhancement (ADE)” in COVID-19 patients provides key insights to develop potential therapeutic interventions for immediate clinical management. Therefore, in this review, we have described the details of rapid detection methods of SARS-CoV-2 using molecular and serological tests and addressed different therapeutic modalities used for the treatment of COVID-19 patients. In addition, the current challenges against the development of vaccines for SARS-CoV-2 are also briefly described in this article.

## 1. Introduction

SARS-CoV-2 infection spreads through the respiratory droplets when an infected person is in close contact with other individuals [1]. To date, there are wide ranges of therapies developed and evaluated for the effective management of COVID-19. For instance, the existing treatment methods such as antiviral drugs (remdesivir), antibodies (intravenous hyperimmunoglobulin therapy), anti-inflammatory drugs (statins, dexamethasone), immunomodulatory therapies, anticoagulants, and antifibrotics are reported to exhibit different therapeutic efficacies during COVID-19 treatment [2, 3]. However, currently, there is no single therapeutic modality proven effective apparently to mitigate this disease progression in hospitalized COVID-19 patients [1].

### 1.1. Structure and Pathophysiology of SARS-CoV-2

**Figure 1:**
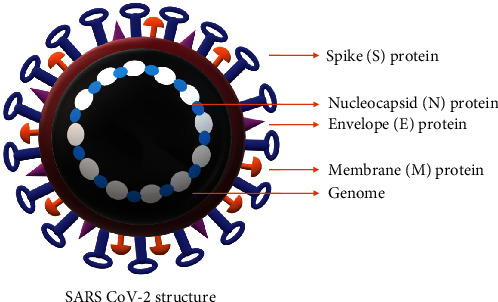
Schematic representation of the structure of SARS-CoV-2: SARS-CoV-2 is an enveloped virus containing RNA genome. The envelope contains spike (S) protein, nucleocapsid (N) protein, envelope protein (E), and membrane protein (M).

Coronavirus exhibits a crown-like appearance due to surface spike (S) glycoproteins when observed under the electron microscope [4]. Coronavirus is composed of a cis-acting RNA genome to foster the viral replication in host cells through RNA-dependent RNA polymerase [5, 6]. Besides, both cis- and trans-acting viral elements participate in spike (S) protein synthesis, coronaviral encapsidation, and packaging into host cells [7]. The spike glycoproteins consist of S1 and S2 heterotrimer subunits, in which S2 subunit significantly conserved with fusion peptide, a transmembrane domain, and a cytoplasmic domain [5] (Figure 1). Mutations in the genes coding for S protein induced the replacement of glycine (G) at 723 positions with serine (S) and isoleucine with proline (P) at 1010 amino acid position. These mutations in S proteins reported were to enhance the invading potential of SARS-CoV-2 [8]. CoV 229E and OC43 strains are detrimental to humans by causing common cold and lower respiratory infections in several immunocompromised patients [9–11]. The coronavirus-induced pathophysiology varies significantly in terms of its impact on alveolar inflammation, neutrophil infiltration, and immune responses during interstitial pneumonia [10, 12–14]. Recent studies have also shown that SARS-CoV-2 infection leads to multiple organ damage, which is due to severe cytokine storm.

## 2. Modes of Transmission of SARS-CoV-2

Current studies have demonstrated that the infected individual can transmit SARS-CoV-2 virus to an average of 2.2 individuals, which is causing a significant increase in the number of individuals suffering from this disease [15]. Even though the virus is reported to be originated in animals and transmitted to humans, the subsequent transmission is primarily through respiratory mode [15]. Respiratory transmission is either by large droplets with virions of a size larger than 5 *μ*m or aerosols smaller than 5 *μ*m expelled out directly from the respiratory tract by the patient. These infectious droplets are reported to remain suspended in the air for an extended period of time and can travel up to 2 to 3 meters distance before they become inactive [16, 17].

Studies have reported a significant reduction in the risk of respiratory transmission of SARS-CoV-2 if a suitable mask is used [18–23]. Studies have shown a fomite or direct contact-mediated transmission of SARS-CoV-2 [24] when patients share common facilities (restrooms, elevators) and follow a poor hand hygiene [25–28]. Furthermore, the vertical transmission of SARS-CoV-2 has been reported in neonates, where three neonates tested positive for IgM on day 2 after birth while the other tested positive within 16 hours after delivery [28, 29]. Although a case report described transplacental transmission of SARS-CoV-2 [30–33], the transmission from breast milk to infants has not been reported yet. Likewise, the transmission of SARS-CoV-2 through the sexual, fecal, oral, and blood also has not been reported.

S proteins are responsible for the viral particle adherence and docking to the human cell surface receptors such as ACE-II [34]. The binding efficacy of S proteins in SARS-CoV-2 to the ACE-II is “10 to 20 times” stronger compared to the binding efficacy of SARS virus reported in 2002; hence, the spread of SARS-CoV-2 from one person to another is much higher and induces the viral-mediated pathophysiology [35]. Despite several structural similarities between SARS-CoV-2 and SARS virus, the antibodies that were effective against SARS failed to neutralize SARS-CoV-2; hence, targeting SARS-CoV-2 using these antibodies is not feasible, which poses a challenging task to medical scientists to develop a potent and specific approach for mitigating SARS-CoV-2 infection [4].

## 3. Need for the Improvement of Existing SARS-CoV-2 Detection Methods

Detection of SARS-CoV-2 is the preliminary step in testing, tracing, treating, and the management COVID-19. Therefore, a sensitive and low-cost screening test is highly essential. The existing SARS-CoV-2 detection tests are broadly divided into (a) molecular methods and (b) serological methods. The molecular methods detect “viral RNA” in the biopsy of nasal tissue (collected using a swab) of the infected individual by using reverse transcription polymerase chain reaction (RT-PCR) (Table 1) [36, 37]. Since the collection of nasal swabs can cause irritation to the soft tissues, studies are currently in progress to check whether saliva could be used to detect SARS-CoV-2 [38]. Andrew Brooks, the Chief Operating Officer (COO) and Director of Technology Development, Rutgers University Cell and DNA Repository (RUCDR) Infinite Biologics, has developed a saliva test, which requires the COVID-19 suspect to spit the saliva in a cup (Table 1). This test has received emergency use authorization approval from the United States Food and Drug Administration (USFDA). Recent studies are encouraging the collection of saliva as diagnostic fluid sample rather than the nasopharyngeal swab for detecting SARS-CoV-2 [38]. USFDA has authorized 22 companies to distribute these testing kits [39]. However, to date, the Indian Council of Medical Research (ICMR), Government of India, has not approved any saliva-based rapid antigen tests for screening and identification of SARS-CoV-2-infected individuals. World Health Organization (WHO) and the Center for Disease Control (CDC) are currently using more advanced RT-PCR-based tests for detecting SARS-CoV-2 accurately. The testing kits developed by Abbott can take about 5 minutes, whereas the rapid testing kits designed by other companies usually require more than 30 minutes to produce reliable detection results [39].

Now, Wyllie and colleagues have examined the possibility of using saliva for diagnosis (salivaomics) and concluded that saliva could be used as an alternative for nasopharyngeal swab [40]. PCR data not only provides an absolute quantification of the number of copies of mRNA but also yields key information about the total viral load for efficient assessment of disease severity [40, 41]. Since appropriate standards are simultaneously subjected to amplification in parallel with saliva, the qPCR is considered as a more precise method to decipher the exact viral load [40]. However, the qPCR test is time-consuming and requires expertise to interpret the results [42].

### 3.1. Serological Tests for the Diagnosis of SARS-CoV-2

Unlike molecular tests, the serological tests can detect the *antibodies* produced against SARS-CoV-2 in the infected or recovered individuals using enzyme-linked immunosorbent assay (ELISA) [43]. The turnaround time (TAT) for serological tests is only 15 minutes; therefore, these diagnostic kits are the preferred choice for the rapid analysis of samples [36]. According to these tests, the presence of IgM indicates “recent exposure”, whereas the presence of IgG indicates “infection in late-stage” [43]. Although serological tests are much easier to execute, they are associated with certain limitations, viz., (a) lack of efficacy to detect the infection at a very early stage due to time gap required to generate antibodies in the body, (b) yielding many false-negative results, and (c) generation of false-positive results if the individual is infected with other related coronaviruses such as HKU1, NL63, OC43, and 229E. Currently, FDA has approved a unique serological test developed by Cellex, USA (Cellex qSARS-CoV-2 IgG/IgM rapid test). Few other companies such as Bodysphere have also announced 2-minute rapid detection methods; however, these tests still require clearance/approval from FDA, USA. The serological test results alone cannot be considered as a confirmatory test, as it requires further validation using molecular tests [39]. Furthermore, the utility of serological tests alone for detecting SARS-CoV-2 is still in debate among medical communities due to their poor accuracy and false positive/negative results [44].

In addition to the above strategies for viral detection, Zhang et al. reported the “CRISPR-Cas13-based SHERLOCK” (specific high sensitivity enzymatic reporter unLOCKing) technique for SARS-CoV-2 diagnosis. The protocol incorporated the Cas13, which targets the S gene, Cas13 enzyme, and ORF1ab gene [45], but this procedure requires further validation using COVID-19 patient samples. Procedurally, the technique involves isothermal amplification of RNA samples using recombinase and polymerase, followed by the incubation of amplified viral RNA with Cas13 enzyme, guide RNA, and reporter. Using a paper dipstick, the distinct band produced by the cleaved reporter is visualized [46].

Council of Scientific and Industrial Research-Institute of Genomics and Integrative Biology (CSIR-IGIB), New Delhi, Government of India, has developed a low-cost “paper-strip based laboratory test” to detect SARS-CoV-2 using CRISPR-Cas9 technology. The test is simple and low-cost (about Rs.500 (five hundred rupees only)) and does not require high-end equipment such as a real-time PCR machine [47]. In addition, this test does not involve the isolation of RNA and conversion of isolated RNA into cDNA and the requirement of PCR reagents etc., which are essential for other molecular testing kits. However, this test requires further validation to establish accuracy and sensitivity and is currently waiting for approval from regulatory authorities in India. At present, a total of 158 RT-PCR kits were validated and approved by the Indian Council of Medical Research (ICMR), Government of India, for screening and testing of SARS-CoV-2-induced COVID-19.

Although the above testing methods could detect the SARS-CoV-2 in infected individuals, the lack of specificity and sensitivity is a major problem and may generate false positive and negative results. Therefore, the prospective research should focus immediately to improve the specificity and sensitivity of SARS-CoV-2 detection methods in clinical samples [36]. One specific approach, which has been gaining medical importance, is the combined detection of SARS-CoV-2 RNA and its viral protein(s) using a signal amplification strategy rather than a “target amplification” procedure. This signal amplification strategy was successfully implemented and demonstrated to be effective in yielding many reliable results in the detection of HPV [48].

### 3.2. Methods of Sampling for Testing and Tracing COVID-19 Patients

Sampling methods do play a crucial role in detecting SARS-CoV-2. Studies have reported the identification of SARS-CoV-2 in respiratory secretions [49–56], feces or rectal swabs [57–61], blood [43, 60, 62–64], oral fluid [40, 65–67], ocular fluids [68–72], urine [73, 74], semen [75], brain tissue [76], and cerebrospinal fluid [77]. Therefore, the choice of sampling method depends on the clinical presentation and the time since the onset of symptoms. Respiratory tissues are the preferred samples to diagnose COVID-19.  Table 2 summarizes various sampling methods used in the detection of SARS-CoV-2.

## 4. Strategies Targeting SARS-CoV-2: Development of Pharmacological Agents to Mitigate and Treat SARS-CoV-2 Infection

**Figure 2:**
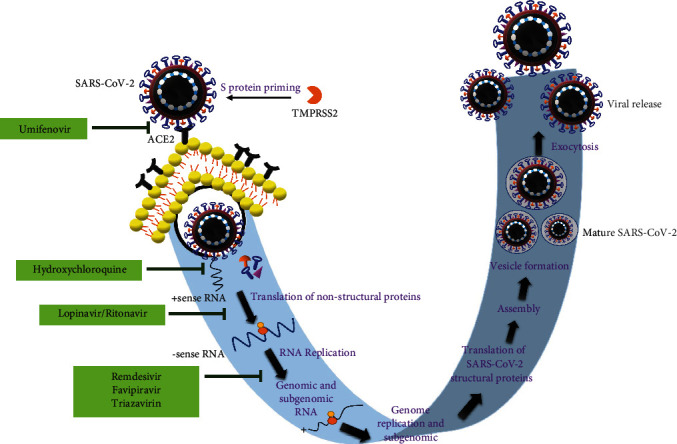
The mechanism of action of umifenovir, lopinavir/ritonavir, remdesivir, favipiravir, triazavirin, and hydroxychloroquine in the treatment of SARS-CoV-2-mediated pathophysiology.

According to WHO, the fatality rate of COVID-19 patients has been increasing across the globe due to lack of selective therapeutic interventions and potent vaccines [96]. Existing vaccines and drug combinations are either selective to a particular variant of the virus or exhibit systemic toxicity. Therefore, it is crucial to develop potent, pan-specific, and long-lasting vaccines and better pharmacological agents for the prevention and treatment of SARS-CoV-2 infections (Table 3). The combination of alpha-interferon and anti-HIV drugs lopinavir/ritonavir has shown minimal success and proven to be toxic in recent studies [97] (Figure 2). Currently, a broad-spectrum antiviral drug remdesivir (developed by Gilead Sciences, Inc.) is being used for the treatment [97]. A recent study demonstrated the efficacy of two lead compounds 11a and 11b *in vitro*, which were synthesized using a structure-based drug design approach to target viral main protease (M^pro^), and reported good pharmacokinetic and safety profile in animals [98]. However, further studies are warranted to consider these drugs for clinical use. Similarly, many other studies have also reported the development of drugs targeting various viral proteins and postinfection events [97, 99].

Decreasing the viral load in infected individuals is one of the main strategies and considered by many investigators to reduce the COVID-19 complications. Supporting this idea, interestingly, the fatality rate was reported to be significantly low in pediatric patients as they have relatively low seroprevalence compared to adults [97, 99, 100]. Favipiravir is a derivative of pyrazinecarboxamide, which acts by inhibiting RNA-dependent RNA polymerase [97]. Favipiravir is available for the treatment of COVID-19 patients at mild-to-moderate phase. Therefore, a preventive strategy using a potent vaccine is urgently required. However, efforts in developing a potent vaccine are still in progress.

The development of a vaccine requires a thorough knowledge about viral surface glycoproteins (in the case of enveloped viruses such as SARS-CoV-2) and capsid proteins (in the case of nonenveloped proteins) [97]. The genome of SARS-CoV-2 encodes both structural (spike—S; membrane—M, envelope—E, and nucleocapsid—N) and nonstructural proteins that play crucial roles in the assembly and rapid spread of virus among the population [101]. SARS-CoV-2, similar to CoV-NL63, can use ACE-II receptors, which is a characteristic antigenic commonality of several coronaviruses with zoonotic potential [100]. ACE-II is extensively expressed in the gastrointestinal tract where viral shedding is marginally prolonged in the stools due to ACE-II binding. Despite extensive similarities between SARS-CoV and SARS-CoV-2 (>90% similarity between SARS-CoV and SARS-CoV-2 N, E, and M proteins and 76% similarity in S proteins), the available knowledge about key immunological epitopes, which are responsible for antibody and T cell responses, is very minimal [101]. Therefore, developing an effective vaccine against SARS-CoV-2 is still a major challenge.

### 4.1. Monoclonal Antibodies (MABs) against SARS-CoV-2

COVID-19 patients are characterized by the presence of a dysregulated immune system, hyperinflammation, and very high IL-6 levels. IL-6 is one of the key cytokines implicated in COVID-19 severity and patient mortality [102–104]. Genomic analysis revealed that critically ill patients with COVID-19 exhibit genetic variations in IL-6-mediated inflammatory pathway proteins, which cause life-threatening disease [105]. The accumulation of lymphocytes, inflammatory monocytes, and other mediators such as apoptotic proteins and thrombotic factors results in pulmonary damage in these patients [106–109]. In addition to vascular permeability, the IL-6 can foster endothelial dysfunction; hence, IL-6 is an attractive drug target for mitigating the complications of COVID-19 [110, 111] (Figure 2). For instance, the administration of tocilizumab to COVID-19 patients resulted in the impaired activity of IL-6*α* receptors, which consequently fostered good clinical outcomes in these patients [112–114]. This was confirmed by several case reports, retrospective observational cohort studies, and randomized clinical trials. According to COVACTA phase 3 clinical trial, tocilizumab efficiently mitigated COVID-19-induced clinical manifestations such as fever and pneumonia [102–105, 115–117].

Sarilumab is another monoclonal antibody reported to be effective against SARS-CoV-2 by inactivating IL-6-mediated acute inflammatory responses [116–118]. It has proven efficacy in mitigating cytokine storm. Clinical usage of sarilumab was further confirmed by enhancement in patient survival and mitigation of multiple organ damage in critically ill patients with COVID-19[119–123].

### 4.2. Baricitinib and COVID-19

Adaptive COVID-19 treatment trial-2 (ACCT-2) has tested the benefit of combining baricitinib (a specific inhibitor of Janus kinase-1 and Janus kinase-2) with remdesivir in critically ill patients of COVID-19. Both primary and secondary clinical outcomes are reported to be satisfactory [124]. In addition, two reports of Cantini et al. (2020) also concluded the efficacy of baricitinib in inducing the impairment of JAK1 and JAK2, which consequently blocked the immune cascades and viral replication [125–127]. However, the supplemental oxygen through mechanical ventilation is an intriguing subject of research in COVID-19 patients who are receiving baricitinib and dexamethasone.

## 5. Challenges in the Development of Vaccines against SARS-CoV-2

Recent studies have shown that T cell responses against viral structural proteins are more immunogenic and long-lasting (up to 11 years of postinfection) when compared to nonstructural proteins and antibodies [101]. In a recent report, Walls et al. (2020) have identified a set of epitopes in S and N structural proteins that can launch an effective response against SARS-CoV-2 [101]. Furthermore, the authors of this study have incorporated significant details about the epitope associated with MHC alleles so that a wide population range can be covered globally [101]. Virus-specific effector memory T cells can encounter coronaviral strains thereby mitigate the complications of infections [135]. In the case of SARS-CoV and MERS-CoV, these viruses can use non- or subneutralizing antibodies and induce immune responses via the antibody-dependent enhancement (ADE), a kind of Trojan horse mechanism [136, 137]. ADE is involved in several viral infections such as Zika virus, Ebola, SARS-CoV, SARS-CoV-2, and HIV [136, 137]. In the case of SARS-CoV-2, the significant immune mechanism occurs via CD32a-mediated ADE, which limits the efficacy of current vaccination [96]. CD32 is an extensively expressed protein on the surface of monocytes and macrophages (ex. alveolar macrophages), which gets aggregated by IgG.

T cell responses are crucial when compared to the humoral responses as these T cell responses have a significant influence on the recovery from primary infection and avoid reinfection [97]. These immune responses can influence vaccine development against COVID-19. The vaccination should enhance both humoral immunity and cellular responses in order to prevent COVID-19-induced complications [138, 139]. In the first phase, the virus infection can be mitigated to reduce initial viral load and control the spread of SARS-CoV-2 to other respiratory organs. In the next phase, the cellular immune responses become significant and help in mitigating the inflammatory phase of COVID-19 disease. In the case of convalescent plasma therapy, the humoral response could be triggered by the vaccine to confer protection against SARS-CoV-2 [97].

### 5.1. Current Status and Challenges for COVID-19 Vaccination

The development of safe and efficacious vaccines against SARS-CoV-2 is a challenging task [4]. Previously, vaccines against coronaviruses were developed using passive/active immunization and used the animal models for SARS-CoV replication. This is due to the nonavailability of authentic animal models for the development of coronavirus vaccines [140–142]. The passive transfer of immune serum can mitigate the SARS-CoV load in naive BALB/c mice [143]. Earlier, Cheng et al. (2005) have reported the efficacy of “SARS-CoV neutralizing antibodies in SARS hyperimmune globulin” isolated from human convalescent-phase plasma for neutralizing the SARS-CoV infection [144]. Human SARS-CoV administration to animal models has been reported to mitigate the outbreaks of coronavirus infection suggesting that future studies should uncover these mechanisms for SARS-CoV-2 infection [4, 145–147]. However, many experimental studies must be conducted to ascertain the activity of MABs for their neutralizing efficacy by analyzing the immune memory repertoire of COVID-19 patients. Even though the usage of antiviral drugs, viz., *proteinase inhibitors*, *calpain inhibitors*, *nucleoside analogues*, *interferons*, and *siRNAs* against SARS-CoV-2 infection, is reported in recent times, several conflicting results with wide variations in clinical outcomes have been generated, which necessitate a global approach for the development of effective vaccines [148–153]. Therefore, a concerted effort is urgently warranted to develop a potent and clinically viable vaccine against SARS-CoV-2 [154].

Reverse genetics technology may be another important strategy to develop vaccines against coronavirus infections [153]. Recent reports have described the efficacy of both mutant and chimeric recombinant viruses to uncover the function of S protein in coronavirus [155, 156]. Reverse genetics has been widely used to elucidate the “structure/function relationship of viral UTRs at 5′ → 3′ of the genome” and the “function of replicase gene for enzymatic activity” in mediating coronaviral replication and pathogenesis [155, 157]. In addition, this strategy is significantly essential to express foreign gene sequences in place of noncoding genes which can help in the development of attenuated vaccines against coronaviruses [158–161].

### 5.2. Antibody-Dependent Enhancement (ADE) and SARS-CoV-2 Vaccines

**Figure 3:**
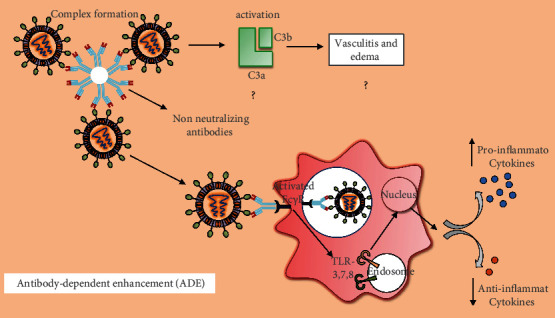
The process of antibody-dependent enhancement (ADE) in lung cells. Entry of SARS-CoV-2, which is mediated by ACE-2 receptors on lung cells, further actuates inflammatory cascades through the production of pathogen-specific antibodies followed by ADE. ADE consequently induces lung pathology through the engagement with Fc receptors expressed on several immune cells, viz., monocytes and macrophages. Internalization of ADE-induced immune cascades can foster inflammation and tissue damage by modulating the inflammatory factors in lung cells.

The extensive immune backfiring induced through ADE is one of the critical reactions associated with SARS-CoV-2 infection. ADE is progressively produced from this viral infection followed by the induction of Th2 immunopathology, which further blocks the attempts to develop a safe and effective vaccine against SARS-CoV-2. ADE can modulate the immune reactions and induce sustained inflammation, lymphopenia, and cytokine storm, which further lead to the severe disease and death. ADE also requires prior exposure to similar antigenic epitopes most likely from the circulating viruses [162]. For instance, the neutralizing antibodies exhibit a greater ability to block viral entry, fusion without additional immune mediators, although the Fc region is mandatory for neutralizing the influenza virus [163]. In the case of SARS-CoV, the viral docking on ACE-2 was potentially impaired by the administration of neutralizing antibodies since they can recognize and block receptor-binding domain (RBD) and heptad repeat 2 (HR2) domain on the spike (S) protein [164]. Neutralizing antibodies could foster the immune activities of phagocytes, complement, and NK cells [162]. SARS-CoV-2-specific antibodies significantly can induce lung pathology through ADE engagement with Fc receptors that are expressed on several immune cells such as monocytes, macrophages, and B cells [165]. This process is predominantly independent of ACE-2 expression, pH, and host membrane proteases. Thus, the internalization of ADE-induced immune cascades can foster inflammation and tissue damage by mitigating the anti-inflammatory cytokines IL-10 and TGF-*β* and enhance the levels of the proinflammatory chemokines CCL2 and CCL3 (Figure 3) [166, 167]. However, the underlying mechanisms associated with ADE-mediated immune reaction in SARS-CoV-2 infection are yet to be investigated for effective vaccine development [136, 165].

### 5.3. Th2 Immunopathology and SARS-CoV-2 Vaccines

**Figure 4:**
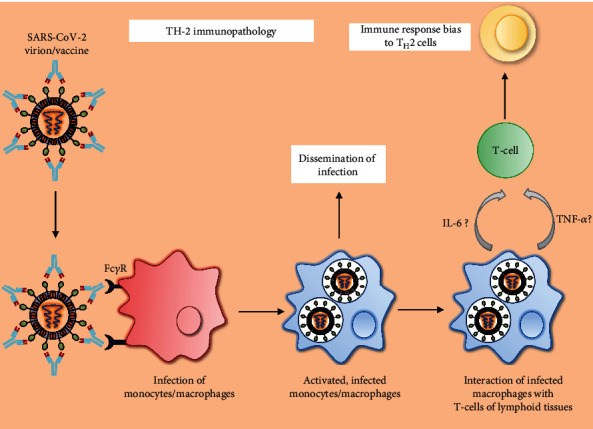
Sequence of events involved in SARS-CoV-2-induced Th2 immunopathology: antibody-bound SARS-CoV-2 virion interacts with the Fc*γ*R of host monocytes/macrophages. Virus-infected macrophages are not only responsible for various complications of the disease but also interact with T cells of lymphoid tissues in the host, which leads to the aggravated inflammatory responses which were reported in COVID-19.

The significant roles of host Th2 immunopathology and Th17 inflammatory responses are responsible for pneumonia and edema in the COVID-19 pathogenesis [168] (Figure 4). Release of IL-17 and granulocyte-macrophage colony-stimulating factor (GM-CSF) exacerbates viral immunopathological events by mitigating Treg cells and enhancing the neutrophil migration with concomitant induction of Th2 responses in the lungs [168, 169]. IL-6-mediated Th17 differentiation can foster lung pathology during SARS infection [170]. Th2-type immunopathology along with eosinophil infiltration has been observed with SARS vaccination against SARS-CoV in mouse models [171]. However, confirmatory studies are yet to be performed for IL-6-mediated Th17 responses during SARS-CoV-2 infection to develop anti-IL-6 monoclonal antibodies as new therapeutic interventions [114, 172]. RBD-based subunit vaccine is expected to be safer compared to other vaccines, which may induce Th2 immunopathology [173].

The attenuated whole virus vaccine may elicit a significant immune response against SARS-CoV-2 because this virus uses ACE-2 receptors to enter into human cells [174]. Another method is to develop a subunit vaccine, which may induce sensitization of the immune system to foster immune response against S protein subunits of SARS-CoV-2 [153, 175]. In addition, recent studies are currently evaluating the efficacy of nucleic acid vaccines against SARS-CoV-2 to combat COVID-19 [176–178].

## 6. Recent Trends in the Development of Vaccines

**Figure 5:**
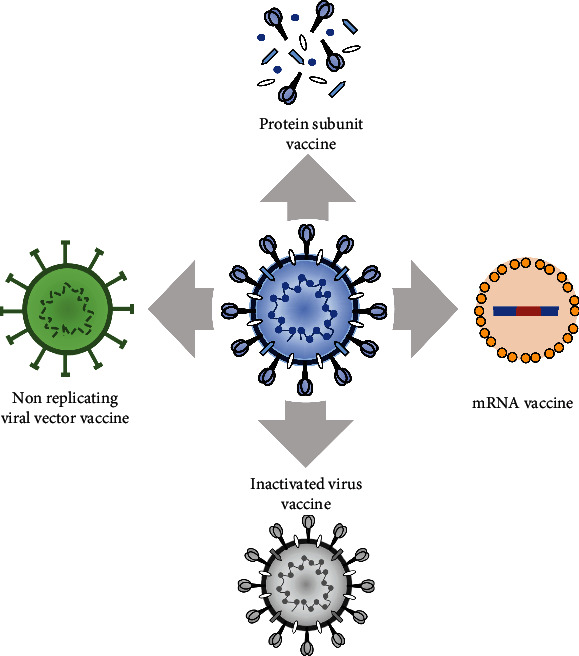
Schematic depiction of the four different kinds of vaccines, viz., nonreplicating viral vector vaccine, mRNA-based vaccine, inactivated virus vaccine, and protein subunit-based vaccine.

As of 13^th^ April 2021, a total of 166 vaccines have been registered; among which, 89 are under clinical trials in humans. A list of vaccines developed against SARS-CoV-2 is given in Table 4 and Figure 5. On 16^th^ January 2021, the COVID-19 vaccines Covishield (Oxford-AstraZeneca and Serum Institute of India) and Covaxin (Bharat Biotech) were launched in India. Initially, these vaccines were made available for healthcare and frontline workers. As of 1^st^ March 2021, these vaccines are also made available for individuals aged above 60 years of age and the ones aged between 45 and 59 years with comorbid conditions like cancer, diabetes, and hypertension. To date, worldwide, approximately 825 million vaccine doses have been administered. However, there is an immediate requirement for safe and effective vaccines as the number of SARS-CoV-2 infected cases is increasing at alarming rates with a current global estimate of 138,027,200 confirmed cases.

### 6.1. mRNA-Based Vaccines

#### 6.1.1. mRNA-1273

The mRNA-1273, an mRNA vaccine, was developed by Boston-based Moderna therapeutics and the National Institute of Allergy and Infectious Diseases (NIAID), USA. The vaccine encodes the prefusion form of the SARS-CoV-2 spike protein in a lipid nanoparticle vector. Upon administration, mRNA undergoes transcription and translation to produce viral antigens. The immune system recognizes these viral antigens and initiates an adaptive immune response against the S protein of SARS-CoV-2. The preclinical studies on BALB/cJ, C57BL/6J, and B6C3F1/J mice showed induction of virus-specific antibodies upon administering intramuscular doses of 1 *μ*g mRNA-1273, 3 weeks apart [179]. The phase 1 trial began on March 16^th^, 2020, with 45 healthy volunteers of age between 18 and 55 years. They were administered with three different doses (25 *μ*g, 100 *μ*g, and 250 *μ*g), and the second dose was after four weeks. The trial reported a strong CD4+ T cell response and produced neutralizing antibodies, while CD8+ T cell responses were recorded by the medium-level dose (100 *μ*g)[180, 181]. Myalgia, fatigue, headache, chills, and pain at the injection site were side effects recorded only after the second dose of vaccination and were prominent only in the group who had received the highest dose (250 *μ*g). In a study of 40 older adults (56-70 years or ≥71 years), the immunogenic response was similar to 18-55 years, indicating its efficacy and less immunocompetency in all age groups. In the phase 2 trial, 300 young and 300 older adults were recruited to determine the ability of 25 *μ*g and 100 *μ*g doses. The data reported significant immunogenic responses at 100 *μ*g dose. Phase 3 trials began on July 27^th^, 2020, with 30,420 participants in the USA, where half of the participants (15,210) received two doses of 100 *μ*g of mRNA-1273 and other half received the placebo [182]. A total of 196 participants have shown symptomatic COVID-19 illness; among which, only 11 participants were vaccinated with mRNA-1273 indicating 94.1% [183] efficacy without any long-term adverse effects. Pain and redness at the injection site, myalgia, arthralgia, headache, and fatigue were the short-term adverse effects reported after the second dose. The vaccine remains stable at 2°C to 8 °C for 30 days, -20°C for 6 months, and room temperature up to 12 hours [184].

#### 6.1.2. BNT162b2

BNT162b2 (Comirnaty®; BioNTech and Pfizer) was developed and manufactured by Pfizer, Biopharmaceutical New Technologies, and Shanghai-based Fosun Pharma. This vaccine has been approved for usage in several countries, viz., Bahrain, Brazil, New Zealand, Saudi Arabia, and Switzerland [185]. In the preliminary trials of this vaccine in BALB/c mice, the effective humoral anti-SARS-CoV-2 immune response was reported without any clinical signs of disease. The immunized mice showed CD8+ and CD4+ T lymphocytes activation and neutralizing antibodies, which was determined by a GFP-encoded vector on the envelope of SARS-CoV-2 [185]. In phase 1 trials, participants of age groups 18-55 years and 65-85 years showed minimum side effects when administered with BNT162b1 and BNT162b2[185]. Even though high-dose-dependent neutralizing antibodies were produced by both the candidates, BNT162b2 reported to produce less reactivity in older adults with a 30 *μ*g dose range; therefore, this vaccine candidate is considered for large-scale phase 2/3 studies [185, 186]. The phase2/3 trial began on July 27^th^, 2020, with 43,488 volunteers. The study participants included individuals with comorbid conditions. The two-dose immunization with 30 *μ*g of BNT162b vaccine induced neutralizing anti-SARS-CoV-2 antibodies and rendered 95% protection against the disease [187]. The most frequent side effects of this vaccine were fatigue and headache [187].

### 6.2. Nonreplicating Viral Vector Vaccine

#### 6.2.1. Ad5-nCoV

The replication-defective vector vaccines, which are under phase 3 trial, are Ad5-nCoV, AZD1222, Sputnik V, and JNJ-78436735 [187]. Ad5-nCoV is a vaccine candidate that encodes the S protein of SARS-CoV-2 into host cells. It was developed by CanSino Biologics and the Institute of Biology of the Academy of Military Medical Sciences (AMMS), China [188]. The preclinical studies on BALB/c mice with a dose of intramuscularly or intranasally injected Ad5-nCoV induced humoral response, which subsequently enhanced the levels of neutralizing antibodies against SARS-CoV-2[188]. Results of phase 1 trial on 108 participants of age group between 18 and 60 showed that doses of 5 × 10^10^ and 1 × 10^11^ viral particles/dose were safe and produced good immunogenicity in study participants [189]. In phase 2 trial with 508 participants of age group 18-83, both the doses showed an equal immune response; however, mild adverse effects were reported in 74% and 72% of participants in the lower and higher dose groups, respectively [190]. The phase 3 trial was initiated in September 2020 with a dose of 5 × 10^10^ viral particles/dose in 40,000 volunteers in Saudi Arabia, Russia, and Pakistan. However, Central Military Commission of China has restricted the use of this vaccine in the military [187].

#### 6.2.2. AZD1222

AZD1222 was developed by Oxford University and AstraZeneca, using a chimpanzee adenovirus (ChAdOx1) vector, which encodes the spike protein of wild-type SARS-CoV-2. In preclinical studies, the intramuscular administration of two doses of AZD1222 to BALB/c and CD1 mice had generated a high immunogenic profile [191]. Subsequent studies have evaluated the efficacy of this vaccine even in pigs. Further analysis using “lentiviral-based SARS-CoV-2 pseudovirus neutralization assay” reported significantly enhanced neutralizing antibodies in the study groups. In the phase 1 study of 1090 healthy volunteers of age group 18-55 years, single or double dose (5 × 10^10^ viral particles/dose) of AZD1222 exhibited no side effects but produced a strong neutralizing responses against the virus [191, 192]. During August 2020, phase 3 trials were initiated with 30,000 participants in the USA, India, Brazil, Russia, and South Africa [193]. However, due to severe neurological symptoms, the phase 3 studies were temporarily halted in September 2020 [193]. In-depth investigations should be performed to delineate prominent causes for such severe neurological symptoms; however, preliminary phase 3 trial concluded that these effects were due to undiagnosed multiple sclerosis at the time of vaccination, but not related to the vaccine [193]. The phase 3 study was resumed in other countries, except the USA. Results of phase 3 reported 70.4% efficiency without any severe effects. The vaccine has been approved for use in the UK on November 27^th^, 2020, in Argentina on December 20^th^, 2020, and in India on January 3^rd^, 2021 [194].

#### 6.2.3. Sputnik V

Gamaleya, a Russian Research Institute, developed an adenoviral vector vaccine named Sputnik V [195]. Results of the phase 1/2 trial, which involved 38 participants, have shown an excellent immunogenic profile with mild side effects, myalgia, arthralgia, fever, headache, and minimal pain at the site of injection [187, 195]. Concerns regarding the vaccine's safety and efficacy were raised, as the Health Ministry approved the Russian Federation's vaccine before phase 3 trials. On September 7^th^, 2020, the phase 3 trial was initiated by recruiting 40,000 individuals across Russia and the Republic of Belarus. After a detailed analysis of 18,794 individuals, 91.4% efficacy was reported from the phase 3 study [195]. However, eight participants were tested COVID-19 positive among the vaccinated group. The vaccine trial has not reported any adverse effects except that some of the individuals experienced mild side effects such as fatigue and headache [187, 195].

#### 6.2.4. JNJ-78436735

It is a replication-defective adenovirus vector (JNJ-78436735) developed by Janssen Pharmaceuticals (Johnson & Johnson) [187]. The vector is engineered for expressing the stabilized prefusion S protein of SARS-CoV-2 in the host. This vaccine was tested in Syrian golden hamsters with a single injection of the vaccine candidate, which resulted in the production of neutralizing antibodies against SARS-CoV-2 and reduced the severity of the disease and mortality. Studies on rhesus macaques reported a significant induction of antibody and T cell-mediated responses. The phase 1/2 trials were initiated in July 2020. Two different doses (0.5 × 10^11^/dose or 1 × 10^11^/dose) were administered to the participants of two different groups. Whereas the first group is composed of 402 individuals aged 18-55 years, the second group consisted of 394 individuals aged 65 years or above. According to this trial report, only mild symptoms such as fever, pain at the injection site, and headache were reported upon administration of this vaccine. Approximately, 80% of vaccinated individuals showed CD4+ T cell responses [187, 196, 197]. Phase 3 studies for this vaccine were initiated in the month of September 2020 with 60,000 individuals. The trial was paused because of the development of severe adverse effects in one of the vaccinated individuals; however, the exact clinical manifestations are not reported. The manufacturer announced the second phase 3 study, which involved recruiting 30,000 adults from Belgium, Colombia, France, Germany, Philippines, South Africa, Spain, United Kingdom, and USA [198].

### 6.3. Inactivated Virus Vaccine

#### 6.3.1. CoronaVac

CoronaVac is an inactivated virus vaccine candidate with alum adjuvant. CoronaVac was developed by Sinovac Research and Development Co. A recent study reported high immunogenic profile of CoronaVac in BALB/c mice and Wistar rats. This vaccine reported to induce the activation of SARS-CoV-2-specific neutralizing antibodies. A study was performed on rhesus macaques to determine the efficacy of this vaccine, and the outcome of this study has concluded complete protection against SARS-CoV-2. The phase 2 study was conducted in 600 healthy participants aged between 18 and 59 years, who had received two different vaccine doses (3 and 6 *μ*g/0.5 ml) [199, 200]. As per this study, subjects in both dosage groups exhibited mild adverse reactions and induced more than 90% seroconversion. In the phase 3 trial, a total of 8870 participants from Brazil, Indonesia, and Turkey were recruited and administered with two vaccine doses (2 weeks interval). Finally, this vaccine has been approved in China [187].

#### 6.3.2. Wuhan Institute of Biological Products and Sinopharm Vaccines

An inactivated virus was isolated from WIV04 SARS-CoV-2 strain from a Jinyintan Hospital patient, Wuhan [201]. This vaccine candidate was developed by the Wuhan Institute of Biological Products and Sinopharm by inactivating the virus with *β*-propiolactone and alum adjuvant adsorption procedure [201]. In the phase 1 trial, three different doses (2.5 *μ*g, 5.0 *μ*g, and 10 *μ*g) were administered to 96 participants of aged 18 to 59 years. In the phase 2 trial, 224 participants were recruited and administered two doses of 5.0 *μ*g each [187]. Administration of the vaccine triggered mild adverse effects but induced the activation of neutralizing antibodies against SARS-CoV-2. This vaccine was approved only in China and in the United Arab Emirates [187, 201].

#### 6.3.3. BBIBP-CorV

BBIBP-CorV is an inactivated virus isolated from 19nCoV-CDC-Tan-HB02 strain of SARS-CoV-2. The vaccine is developed by Sinopharm and the Beijing Institute of Biological Products [202]. Studies on animal models demonstrated the production of neutralizing antibodies against SARS-CoV-2 and rendered protection against SARS-CoV-2 [202, 203]. In the phase 1 study, a total of 192 participants were recruited and administered with one of the three different doses of the vaccine (2.0 *μ*g, 4.0 *μ*g, or 8.0 *μ*g). Fever in 10% of participants was observed as an adverse effect. In the phase 2 trials, a total of 448 participants were recruited and administered either one dosage of 8.0 *μ*g or two doses of 4.0 *μ*g of vaccine at 2, 3, or 4 weeks apart [202]. Results of this trial showed a high immunogenicity and excellent safety profile with 4.0 *μ*g/dose with an interval of 3 weeks. Furthermore, the vaccine has been examined for its efficacy in a phase 3 trial in Argentina, Bahrain, Jordan, Egypt, and UAE., among 63,000 participants. Two doses of vaccine (4.0 *μ*g) in 3 weeks were administered. The vaccine was approved for public administration in UAE, Bahrain, China, and Egypt [202].

#### 6.3.4. Covaxin

Covaxin is developed by India-based Bharat Biotech. and the Indian Council of Medical Research, Government of India. It is an inactivated virus developed in Vero CCL-81 cells [204]. The vaccine is isolated from an Indian strain inactivated by *β*-propiolactone along with the alum and imidazoquinoline adjuvant adsorption procedure. The inactivated whole SARS-CoV-2 virion was absorbed into the alum and Algel. This vaccine exhibited a significant reduction in the viral loads and bronchoalveolar infection in animal models [204]. Later, the phase 1 trial was conducted on 375 participants with three different formulations and two different dosages (3.0 *μ*g or 6.0 *μ*g). The vaccine showed a high safety and immunogenicity profile with mild-to-moderate adverse effects with a seroconversion rate of 93.4% in the 3.0 *μ*g dosage group [205]. The phase 3 trials were launched on October 23^rd^, 2020, in India, with a total of 26,000 study participants of different age groups. The participants have received a vaccine dosage of 3.0 *μ*g in the adjuvant formulation and compared with control groups in the study (2 doses/4 weeks apart). The use of this vaccine has been approved in India and currently in usage for the vaccination of the general public [205].

### 6.4. Protein Subunit Vaccine

#### 6.4.1. NUX-CoV2373

NUX-CoV2373 is a protein subunit vaccine developed by Novavax. It is a recombinant SARS-CoV-2 S glycoprotein nanoparticle in a baculovirus-Sf9 vector with an adjuvant Matrix M1 [206, 207]. The adjuvant vaccine formulation was investigated in animal models (BALB/c mice), and it has shown a significant increase in the antibody production and strong T cell response [207]. In the phase 1/2 trial, 131 healthy participants received a total of two doses of the vaccine with and without adjuvant. The results of this trial reported a significant increase in the “immune response with vaccine”. Furthermore, anti-S IgG and neutralizing antibody levels were comparatively higher in the vaccinated subjects than those in the convalescent sera of COVID-19 patients [208]. First phase 3 trial was launched on September 23^rd^, 2020, with 9000 participants in the United Kingdom. Second phase 3 trial of this vaccine was initiated in the US with 30,000 participants in collaboration with Serum Institute of India. The study participants have been receiving a vaccine dosage of 5.0 *μ*g dose with 50 *μ*g of Matrix M1 adjuvant. Results of this study are yet to be announced [208].

## 7. COVID-19 Vaccines: Choices in a Crisis

Despite having a viable vaccine, the search for a more potent and pan-SARS-CoV-2-specific vaccine continues as the virus is capable of acquiring mutations and exhibits significantly variable pathophysiological effects. For instance, in a recent report, a total of 771 variants of SARS-CoV-2 were identified in India. Moreover, several hurdles, concerns, and queries regarding the safety and quality of the vaccines still persist, for example, (a) the demand for a vaccine against the COVID-19 pandemic far exceeds the supply. Hence, there is a shortage of vaccines to meet the increasing demand; (b) confusion about the safety and efficacy of existing vaccines. In this regard, the efforts have to be made to increase the percolation of information to the public [209, 210]; (c) the feasibility of producing and transporting the vaccine as per the requirement in several countries; (d) whether the vaccine is safer to administer to the “pregnant women and the individuals suffering from chronic health complications such as heart diseases”; and (e) the feasibility of supplying the vaccine at free of cost or at affordable price for the general public. Therefore, further studies are warranted to address all these queries, which will help to understand more about the vaccine and encourage individuals to attend vaccination camps for timely vaccination.

## 8. Safety Concerns Pertaining to COVID-19 Vaccines

In general, vaccination is known to induce minimal and transient side effects such as antigen-antibody-mediated reactions, urticaria, fever, and rare skin reactions, which may subside without any major interventions. However, questions about the safety of vaccines arise when the adverse events become a major health concern. Clinical trials of the BNT162b2 mRNA COVID-19 vaccine have been reported to cause minimal local and systemic side effects, viz., pain, redness, fatigue, joint pain, and muscle pain within 1 to 2 days of vaccination [211]. The excipients such as polyethylene glycol (PEG) derivatives may trigger mild anaphylactic adverse reactions upon administration [212]. Anaphylaxis is a serious adverse reaction that can foster asphyxiation, cardiovascular collapse, and multiorgan dysfunction, and sometimes may lead to death [212]. Therefore, it is crucial to delineate prompt recognition of these adverse effects. In general, the anaphylactic reactions are mediated by the mast cell activation via antigen binding and IgE cross-linking. Consequently, these events could trigger the tissue generation of inflammatory mediators such as histamines, prostaglandins, and leukotrienes and foster the development of hives, tachycardia, hypotension, and cardiovascular collapse. Tryptase is higher in blood at the time of both IgE-mediated anaphylaxis and non-IgE-mediated anaphylaxis. The characteristic release of tryptase from mast cells is indicative of the release of inflammatory mediators through mast cells [213]. These effects have raised concerns about the potential adverse risks after vaccine administration in a public community [214]. Therefore, appropriate measures should be taken to minimize these side effects. Further, proper education and awareness about vaccines should be provided to address these concerns and queries associated with vaccine safety and efficacy.

## 9. Conclusions and Future Directions

SARS-CoV-2 can actuate both innate and adaptive immunities in humans. The uncontrolled inflammatory cascades and blockade of adaptive immune function could induce lung tissue damage at local and systemic levels. Moreover, a drastic decline in the levels of CD4+ T cells, CD8+ T cells, B cells, monocytes, eosinophils, and natural killer (NK) cells was observed in COVID-19 patients. Significant improvement has been observed in the early detection of SARS-CoV-2 in the infected patients due to the development of new serological testing and diagnostic methods. Furthermore, the vaccine development using immunological approaches to block viral entry and replication is associated with a significant limitation of SARS-CoV-2-induced immunopathology. Although the mRNA-based and DNA-based vaccines and protein subunit-based vaccines have been developed, the COVID-19-induced immunopathological changes such as *antibody-dependent enhancement* (ADE) and *Th2 immunopathology* have significant implications in developing suitable antiviral agents. Hence, these aspects should be considered while designing a potent vaccine. Furthermore, studies should also focus on developing a drug-antibody conjugate, which can bind to the viral proteins while mitigating the exacerbations in already infected individuals.

## Figures and Tables

**Table 1:** List of rapid methods used for detecting SARS-CoV-2.

Diagnostic tests	Mechanism	Sample	Advantages	Limitations
Direct tests				
RT-PCR	SARS-CoV-2-specific hybridization probes are used to target envelope (E), RNA-dependent RNA polymerase (RdRp), and ORF1b and N regions of the virus. This test can detect the virus at least after two days after infection	Upper respiratory tract (URT) and lower respiratory tract (LRT) specimens	This test is a gold standard method for the diagnosis in symptomatic and asymptomatic patients. This test has a high sensitivity (~89%) and specificity (99%)	Needs infrastructure, very expensive, and requires qualified personnel
Reverse transcription loop-mediated isothermal amplification	Exponential amplification of virus-specific genes at a constant temperature	URT and LRT specimens	High sensitivity and specificity	Needs infrastructure, very expensive, and requires trained personnel
Nucleoprotein (NP) antigen detection test	Enzyme-linked immunoassay has a microplate precoated with specific antibodies against SARS-CoV-2 NP and the use of horseradish peroxidase- (HRP-) labeled secondary antibody	URT and LRT specimens and saliva	Simple and rapid technique. No trained personnel and expensive laboratory instruments are required	Less sensitivity (70-86%) and specificity (95-97%) when compared to RT-PCR
Indirect tests				
ELISA	Detects anti-SARS-CoV-2 IgG and IgM by identifying antibodies against the NP and spike proteins	Serum, plasma, whole blood	Widely used technique, inexpensive, easy sample collection, and high sensitivity (~82%) and high specificity (97%)	Needs infrastructure and trained personnel
Chemiluminescent immunoassay	Light-producing chemical reactions estimate the titers of IgG and IgM by the amount of the emitted luminous signal	Serum, plasma, whole blood	High-throughput and sensitive (77.9%) technique	Needs infrastructure and trained personnel
Rapid detection kits	Device with colloidal gold-labeled SARS-CoV-2 recombinant protein and murine anti-human IgG antibodies	Fingerpick blood samples	No need of infrastructure, easy sample collection results in 10-15 min	Low sensitivity (~88.6%) and specificity (~90.63%)

Even though several detection methods have been developed, RT-PCR is considered as the gold standard for detection of SARS-CoV-2. The details presented in the table show various diagnostic approaches that have been developed in the detection of SARS-CoV-2.

**Table 2:** List of various sampling methods currently in the usage for SARS-CoV-2 detection.

Type of specimen used for COVID-19 testing	Stage of sample collection	Description
Upper respiratory specimens: nasopharyngeal and oropharyngeal swabs	Early-stage infections (asymptomatic or mild cases)	Individual nasopharyngeal swabs are reported to be more reliable [49, 60, 78, 79]. Combining nasopharyngeal and oropharyngeal swabs increases sensitivity and reliability for detecting COVID-19 [79–82]
Lower respiratory specimens: sputum, endotracheal aspirate, bronchoalveolar lavage	Later in the course of the disease, the individuals with strong clinical suspicion of COVID-19 test negative with URT sampling [56, 60, 80, 83]	Sputum is not recommended because of an increase in aerosol transmission [84]. Requires consultation by a physician. Invasive sampling method
Oral fluid collection methods (i) Posterior oropharyngeal fluid/saliva (spitting/drooling) (ii) Collection of oral fluid using pipette or sponges (iii) Gargling with saline solutions	Individuals with clinical symptoms tested negative for URT	Less invasive and lower risk of exposure to other upon collection, when compared with the collection of URT specimens, therefore suitable for mass screening But not recommended by WHO as the sole specimen type for routine clinical diagnosis [85–88]
Serum specimens	One collected in the acute phase and the other in the convalescent phase (2-4weeks)	Considered when nucleic acid amplification tests negative
Fecal specimens	Second week after the onset of symptoms	Considered when there is clinical suspicion of COVID-19, but URT and LRT are negative [89]
Postmortem specimens (postmortem swabs, needle biopsy, or tissue specimen)	Collected during autopsy	For pathological and microbiological testing [89–95]

URT: upper respiratory tract; LRT: lower respiratory tract; WHO: World Health Organization.

**Table 3:** Key molecular targets of pharmacological agents tested against SARS-CoV-2.

Drugs	Target	Description
Remdesivir	RNA-dependent RNA polymerase enzyme	Used in the treatment of individuals with mild-to-moderate COVID-19 [128, 129] Inhibit viral RNA synthesis It did not reduce mortality, the need for mechanical ventilation, or the duration of hospital stay
Tocilizumab	Interleukin-6 (IL-6)	Used in the treatment of severe cytokine release syndrome In COVID-19 patients, it reduces the use of mechanical ventilation and improves lung function [130, 131] More clinical validations are required [131]
Hydroxychloroquine	Target the binding of S protein to ACE2 receptor [132]	HCQ did not effectively prevent COVID-19 infections as it could not slow down the disease progression, pneumonia, acute respiratory distress, and death
Lopinavir/ritonavir	3CLpro-CoV protease cleaves polyproteins during viral replication and assembly	The combination is used in the treatment of mild, moderate, and severe COVID-19 infection by suppressing the viral load [128] More clinical validations are required
Favipiravir	RNA-dependent RNA polymerase enzyme	Inhibits viral RNA synthesis; more clinical validations are required
Triazavirin	RNA-dependent RNA polymerase enzyme	Inhibits viral RNA synthesis; more clinical validations are required
Umifenovir	Blocks the viral entry to the host	Showed no effect in reducing viral load in COVID-19 patients
Corticosteroids—dexamethasone	Proinflammatory genes coding cytokines, chemokines, cell adhesion molecules, inflammatory enzymes, and receptors [129, 133]	Recommended for patients with severe COVID-19; reduces lung inflammation, duration of mechanical ventilation, and mortality [134], but not recommended to the patients comorbid with diabetes due to the chances of mucormycosis (black fungus) growth

**Table 4:** Current stage of vaccines and their manufacturer.

Types of vaccine	Vaccine name	Phase	Manufacturer	Country of origin
mRNA vaccine	mRNA1273	Phase 3	Moderna	US
	Comirnaty	Phase 2/3	Pfizer-BioNtech	Multinational
Protein subunits	EpiVacCorona	Phase 3	Vector Institute	Russia
	NUX-CoV2373	Phase 3	Novavax	Australia
Inactivated virus	BBIBP-CorV	Phase 3	Sinopharm	China
	CoronaVac	Phase 3	Sinovac	China
	Name not announced	Phase 3	Sinopharm-Wuhan	China
	Covaxin	Phase 3	Bharat Biotech	India
DNA-based vaccine	Convidecia	Phase 3	CanSino	China
	JNJ-78436735	Phase 3	Johnson & Johnson	The Netherlands, US
	Sputnik V	Phase 3	Gamaleya	Russia
	Covishield (AZD1222)	Phase 2/3	Oxford-AstraZeneca	UK

## Additional Points

*Human and Animal Rights*. No animal or human was used in this study. All data were collected from open scientific and public sources.
